# AutoRadiomics: A Framework for Reproducible Radiomics Research

**DOI:** 10.3389/fradi.2022.919133

**Published:** 2022-07-07

**Authors:** Piotr Woznicki, Fabian Laqua, Thorsten Bley, Bettina Baeßler

**Affiliations:** Department of Diagnostic and Interventional Radiology, University Hospital Würzburg, Würzburg, Germany

**Keywords:** radiomics, radiology, machine learning, reproducibility, workflow, image analysis

## Abstract

**Purpose:**

Machine learning based on radiomics features has seen huge success in a variety of clinical applications. However, the need for standardization and reproducibility has been increasingly recognized as a necessary step for future clinical translation. We developed a novel, intuitive open-source framework to facilitate all data analysis steps of a radiomics workflow in an easy and reproducible manner and evaluated it by reproducing classification results in eight available open-source datasets from different clinical entities.

**Methods:**

The framework performs image preprocessing, feature extraction, feature selection, modeling, and model evaluation, and can automatically choose the optimal parameters for a given task. All analysis steps can be reproduced with a web application, which offers an interactive user interface and does not require programming skills. We evaluated our method in seven different clinical applications using eight public datasets: six datasets from the recently published WORC database, and two prostate MRI datasets—Prostate MRI and Ultrasound With Pathology and Coordinates of Tracked Biopsy (Prostate-UCLA) and PROSTATEx.

**Results:**

In the analyzed datasets, AutoRadiomics successfully created and optimized models using radiomics features. For WORC datasets, we achieved AUCs ranging from 0.56 for lung melanoma metastases detection to 0.93 for liposarcoma detection and thereby managed to replicate the previously reported results. No significant overfitting between training and test sets was observed. For the prostate cancer detection task, results were better in the PROSTATEx dataset (AUC = 0.73 for prostate and 0.72 for lesion mask) than in the Prostate-UCLA dataset (AUC 0.61 for prostate and 0.65 for lesion mask), with external validation results varying from AUC = 0.51 to AUC = 0.77.

**Conclusion:**

AutoRadiomics is a robust tool for radiomic studies, which can be used as a comprehensive solution, one of the analysis steps, or an exploratory tool. Its wide applicability was confirmed by the results obtained in the diverse analyzed datasets. The framework, as well as code for this analysis, are publicly available under https://github.com/pwoznicki/AutoRadiomics.

## Introduction

Over the past decades, the search for novel, quantitative imaging biomarkers has been an emerging topic in the research landscape, with the ultimate goal of leveraging the full potential of medical imaging and enabling more personalized medical care (1, 2). Within this field, radiomics has been identified as a potential way to mathematically extract clinically meaningful quantitative imaging biomarkers (so-called features) from medical images of different modalities (3–5). Combined with machine learning (ML), radiomics classifiers have been shown to accurately predict the diagnosis (6), prognosis (7), mutational status / genetic subtypes (8–10), histopathology (8), surgery (11), or treatment response (12). Consequently, there is a huge interest in the clinical and research field to translate the diagnostic and prognostic potential of radiomics to clinical patient care.

This interest has resulted in a large number of scientific publications being issued with a similarly large variety of methods and radiomics pipelines. Besides the inherent issue of model overfitting, which comes with any ML and big data application where the number of features usually considerably exceeds the number of samples in the training set, most radiomics studies also have been proven difficult to reproduce and validate. This may be also due to the large variety of methodology and the lack of an open-science mindset within the research community, with the datasets and code rarely published alongside the results.

Fortunately, an evolving body of open-science frameworks has been accumulating in recent years, and new initiatives aiming at standardization and reproducibility of different aspects of radiomics analysis and ML have been founded. For example, the Image Biomarker Standardization Initiative (IBSI) (3) has addressed the standardization of the radiomic feature extraction process, while the Workflow for Optimal Radiomics Classification (WORC) (2) has been developed in order to automate and standardize a typical radiomics (and ML) workflow.

Performing reproducible radiomics studies usually requires programming skills, since the most prevalent tools in the research community are written in Python language (1–3). This makes it very difficult for clinicians (who will be the ones responsible for clinical translation of trained models and classifiers) to perform radiomics studies by themselves or to simply “play around” with the data.

The aim of this study was to present an intuitive, open-source framework with an interactive user interface for reproducible radiomics workflow. We evaluated its performance on eight publicly available datasets covering varying clinical applications to prove that the framework is able to reproduce previously published studies. AutoRadiomics provides tools for every step of the radiomics workflow (including image segmentation, image processing, feature extraction, classification, and evaluation) with the ability to adjust each step of the workflow as needed. We believe this framework may help to bridge the gap from programmers to clinicians and enable them to quickly experiment with their datasets in a reproducible way.

## Materials and Methods

This analysis is divided into two main parts: Section Framework describes design principles that we followed while designing AutoRadiomics, and Section Experiment provides information on experiments that were performed to evaluate its performance in publicly available tomography imaging datasets.

### Framework

**Figure 1:**
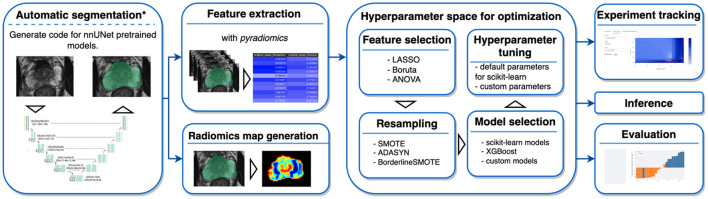
Framework components. AutoRadiomics has a modular architecture, and its components are based on the typical steps in a radiomics analysis. ^*^The first analysis step, automatic segmentation, is not performed inside the framework directly, but a script is generated that can be run separately.

AutoRadiomics is an open-source Python package with an embedded web application with an interactive user interface. The framework can be accessed at https://github.com/pwoznicki/AutoRadiomics, where all the details on its development can be found. The framework is built around the standard steps of a radiomics workflow, including image processing, feature extraction, feature selection, dataset rebalancing, ML model selection, training, optimization, and evaluation. The main components of the framework are presented schematically in Figure 1. AutoRadiomics uses standard libraries validated in multiple radiomics studies, such as *pyradiomics* (1) for feature extraction, *scikit-learn* (13) for ML models and data splitting, and *imbalanced-learn* (14) for over-/undersampling. These reliable building blocks further contribute to creating robust and reproducible workflows. The framework is available under the Apache-2.0 License.

#### Data Preparation and Radiomic Feature Extraction

Data splitting in AutoRadiomics is performed on provided case IDs. Depending on dataset size and application, user can choose to split the data into k folds for cross-validation (with or without a separate test set) or into training/validation/test sets. Radiomic features, including standard shape, intensity, and texture features, are extracted with *pyradiomics*, with additional parameters specified in the parameter file. A few built-in options are provided for this purpose, including extraction parameters validated in previous studies (12, 15). Additional optimizations in computing resource allocation make the extraction process more efficient.

#### Hyperparameter Optimization and Experiment Tracking

Hyperparameter optimization is performed using the Optuna framework (16), which dynamically constructs the search space for hyperparameters and automatically chooses optimal ones. The framework simultaneously optimizes the choice and hyperparameters of the ML classifiers as well as feature selection and oversampling methods, which greatly simplifies the training workflow. The following classifiers are included: logistic regression, support vector machines, random forest, and extreme gradient boosting (XGBoost). Experiments are tracked using an integrated MLFlow tracking dashboard, which allows the user to explore the training artifacts as well as the metrics during and after the training process.

#### Web Application

**Figure 2:**
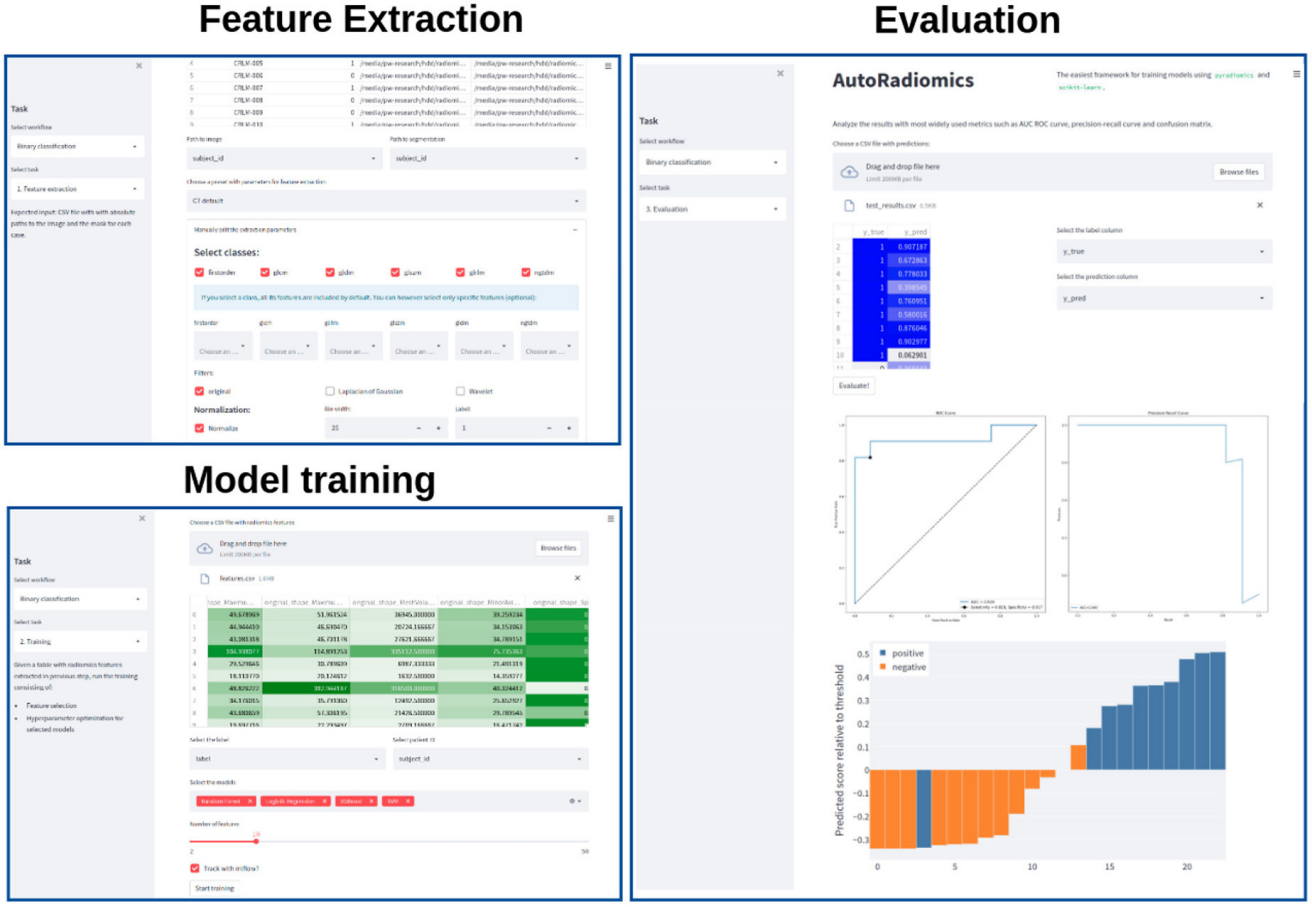
Exemplary screenshots of the web application. The application enables users to perform all the analysis steps including feature extraction, model training, and evaluation, using standardized or custom settings.

Recognizing the problems many non-expert users may face when being forced to use a programming interface, we developed a browser application with an interactive user interface on top of the Python package. The app can be run locally as a Docker container, satisfying the necessary privacy concerns. It adopts a straightforward, modular structure to the radiomics workflow and covers sequentially all steps of the analysis pipeline. The output of each intermediary step, training parameters, and logs are stored in the experiment's directory. That enables the user to later come back to the experiment and document the workflow. Figure 2 presents exemplary screenshots of the app. The app also provides utilities for generating Python code that can be then executed as a separate script to perform automatic segmentation using the state-of-the-art *nnU-Net* framework (17), and for generating radiomics maps using voxel-based feature extraction.

### Experiment

#### Data Sources

To validate the developed framework, we used eight datasets from two different sources. Firstly, we used six public datasets from the recently published WORC database (18), which includes multi-institutional annotated CT and MRI datasets with varying clinical applications. The respective classification tasks were (1) well-differentiated liposarcoma vs. lipoma, (2) desmoid-type fibromatosis vs. extremity soft-tissue sarcoma, (3) primary solid liver tumor, malignant vs. benign, (4) gastrointestinal stromal tumor (GIST) vs. intra-abdominal gastrointestinal tumor, (5) colorectal liver metastases vs. non-metastatic tumor, and (6) lung metastases of melanoma vs. lung tumor of different etiology. The database was released together with benchmark results to facilitate reproducibility in the radiomics field and, to our knowledge, we are the first ones to replicate the previously published results (2).

Additionally, two public prostate MRI datasets, which are available on The Cancer Imaging Archive, were used: Prostate MRI and Ultrasound With Pathology and Coordinates of Tracked Biopsy (19) from the University of California, Los Angeles (UCLA) (further referred to as Prostate-UCLA) and PROSTATEx (20) with annotations from Cuocolo et al. (21). These two datasets were selected since they both had segmentations of prostate gland and lesions as well as biopsy evaluation including Gleason Score (GS) available. All lesions from the Prostate-UCLA dataset had targeted biopsy performed. For PROSTATEx, all lesions with PI-RADS ≥3 were biopsied. We trained radiomics models based on either the whole prostate gland or the target lesion masks in T2-weighted MR images to differentiate between benign prostate lesions and prostate cancer, as well as between clinically significant and clinically insignificant prostate cancer.

#### Data Processing

**Figure 3:**
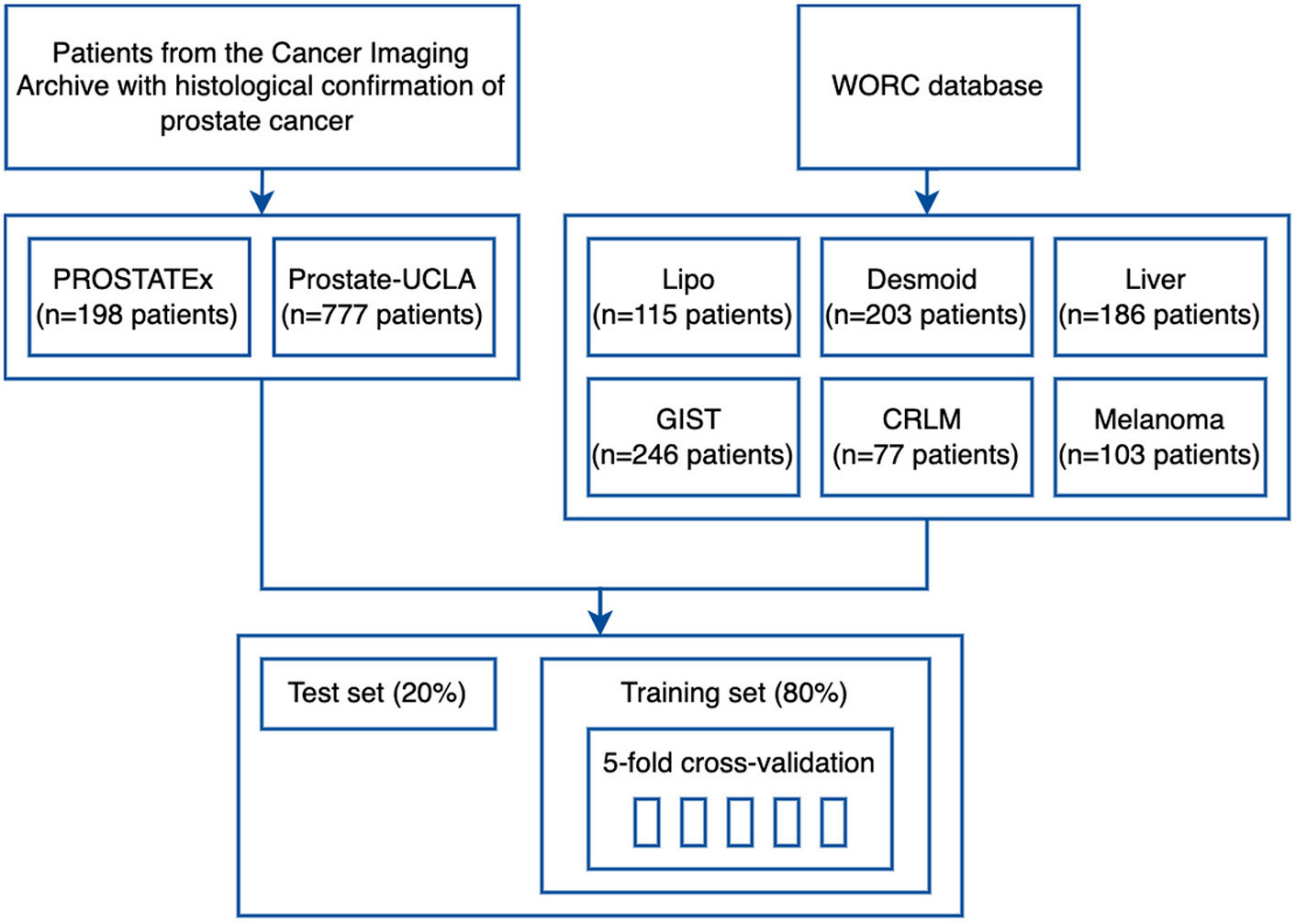
Study flowchart.

The study flowchart is presented in Figure 3. For each dataset, we split 80% of the data into training and 20% into the test set. Then, we split the training set into 5 folds to perform hyperparameter optimization using a cross-validation approach. Image and segmentation data were converted into the NIfTI format, where necessary, and no additional image preprocessing was applied. For feature extraction, we used separate extraction and image processing parameter sets for MRI and CT datasets, as recommended by the IBSI (3). Hyperparameter optimization was performed for each dataset with Optuna using 200 trials of the Tree-structured Parzen Estimator (TPE) algorithm to maximize the objective function.

#### Statistical Analysis

Receiver operating characteristic (ROC) curves were generated for each independent variable and the area under the curve (AUC) was calculated. The diagnostic efficacy of the model was additionally evaluated using the F1 score, sensitivity, and specificity, and was reported with 95% confidence intervals (95% CI) obtained with the bootstrap technique. The bootstrap used 1,000 resamples (with replacement) of predicted probabilities to determine the 95% CI. All analyses were performed with the AutoRadiomics framework, using Python 3.8.10.

## Results

All the experiments were successfully implemented using Python, but can also be reproduced using the interactive web application. Supplementary Figure S1 shows the code extract required to run the optimization and evaluation process for a selected dataset (the implementation assumes a table with data paths is already created). The optimal configurations of models selected for each task are presented in Supplementary Appendix S3. The execution time of the whole pipeline, including the optimization, took around 1 h on a machine with 16 GB RAM and 8-core AMD Ryzen 5800X processor.

The details of training and test cohorts for each task are shown in Table 1. In total, we included 1895 patients in our analyses. In the six datasets from the WORC database, the class distribution was approximately balanced. For the two prostate datasets, the distribution of classes differed between datasets: in PROSTATEx, 50% of index lesions were classified as benign, 15% as GS 6, and 35% as GS ≥7, compared to only 23% of index lesions classified as benign, 24% as GS = 6, and 54% as GS≥7 in Prostate-UCLA.

**Table 1:** Characteristics of training and test cohorts.

		**Number of patients**	
	**Imaging modality**	**Training**	**Test**
Lipo	T1w MRI		
well-differentiated liposarcoma		45 (49%)	11 (48%)
lipoma		46 (51%)	12 (52%)
Desmoid	T1w MRI		
desmoid-type fibromatosis		57 (35%)	15 (37%)
extremity soft-tissue sarcoma		105 (65%)	26 (63%)
Liver	T2w MRI		
malignant primary solid liver tumor		75 (51%)	19 (50%)
benign primary solid liver tumor		73 (49%)	19 (50%)
GIST	CT		
gastrointestinal stromal tumor		99 (51%)	25 (51%)
other intra-abdominal tumors		97 (49%)	24 (49%)
CLRM	CT		
colorectal liver metastases		29 (48%)	8 (50%)
other colorectal tumors		32 (52%)	8 (50%)
Melanoma	CT		
lung metastases of melanoma		38 (50%)	9 (47%)
other lung tumors		38 (50%)	10 (53%)
PROSTATEx	T2w MRI		
benign prostate lesion		80 (51%)	20 (50%)
ISUP grade 1 (GS = 6)		23 (15%)	6 (15%)
ISUP grade 2 (GS ≥ 7)		55 (35%)	14 (35%)
Prostate-UCLA	T2w MRI		
benign prostate lesion		142 (23%)	36 (23%)
ISUP grade 1 (GS = 6)		146 (24%)	37 (24%)
ISUP grade ≥ 2 (GS ≥ 7)		333 (54%)	83 (53%)

*T1w, T1-weighted; T2w, T2-weighted; ISUP, International Society of Urological Pathology; GS, Gleason score*.

**Figure 4:**
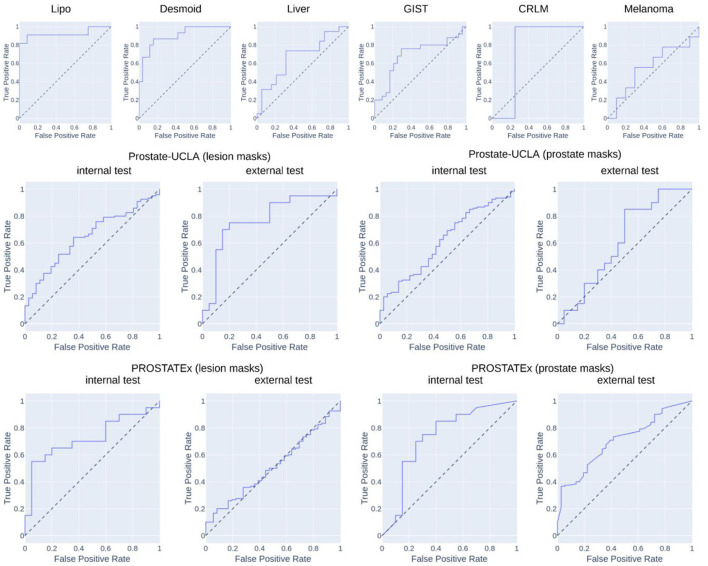
Results of ROC analysis.

Table 2 summarizes the classification results and Figure 4 presents the corresponding ROC curves for all included datasets. In the following, we report the results from the test cohorts.

**Table 2:** Classification results.

**Dataset**	**AUC**		**F1**	**Sensitivity**	**Specificity**
	**Five-fold CV**	**Test**	**Test**	**Test**	**Test**
Lipo	0.86 ± 0.10	0.93 [0.77–1.0]	0.85 [0.67–1.0]	0.82 [0.56–1.0]	0.92 [0.73–1.0]
Desmoid	0.78 ± 0.05	0.90 [0.79–0.98]	0.77 [0.57–0.92]	0.80 [0.58–1.0]	0.84 [0.68–0.96]
Liver	0.64 ± 0.11	0.67 [0.49–0.84]	0.68 [0.48–0.82]	0.69 [0.45–0.88]	0.68 [0.47–0.88]
GIST	0.68 ± 0.03	0.69 [0.53–0.84]	0.72 [0.56–0.84]	0.72 [0.54–0.88]	0.71 [0.5–0.88]
CRLM	0.68 ± 0.14	0.75 [0.43–1.0]	0.82 [0.57–1.0]	0.88 [0.6–1.0]	0.75 [0.43–1.0]
Melanoma	0.59 ± 0.15	0.56 [0.29–0.93]	0.48 [0.15–0.76]	0.44 [0.13–0.8]	0.70 [0.42–1.0]
**Prostate masks**					
PROSTATEx	0.70± 0.03	0.73 [0.55–0.89]	0.73 [0.55–0.86]	0.75 [0.53–0.94]	0.69 [0.47–0.88]
UCLA	0.48 ± 0.09	0.61 [0.51–0.71]	0.78 [0.72–0.74]	0.75 [0.67–0.82]	0.44 [0.28–0.61]
PROSTATEx → UCLA^*^		0.70 [0.62–0.79]	0.52 [0.43–0.62]	0.36 [0.27–0.45]	0.97 [0.90–1.0]
UCLA → PROSTATEx		0.60 [0.41–0.79]	0.69 [0.52–0.83]	0.80 [0.61–0.95]	0.49 [0.27–0.73]
**Lesion masks**					
PROSTATEx	0.68 ± 0.10	0.72 [0.56–0.86]	0.68 [0.48–0.84]	0.55 [0.33–0.76]	0.95 [0.82–1.0]
UCLA	0.59 ± 0.03	0.65 [0.55–0.74]	0.73 [0.67–0.80]	0.64 [0.55–0.73]	0.64 [0.48–0.8]
PROSTATEx → UCLA		0.51 [0.41–0.61]	0.31 [0.21–0.41]	0.19 [0.12–0.27]	0.92 [0.82–1.0]
UCLA → PROSTATEx		0.77 [0.60–0.91]	0.74 [0.57–0.87]	0.70 [0.5–0.89]	0.80 [0.61–0.95]

*Results are presented as mean ± std for five-fold cross validation and mean with 95% CI in brackets for test set*.

* *arrow denotes external validation of the model trained on PROSTATEx in the Prostate-UCLA dataset*.

*FIve-fold CV, five-fold cross-validation; UCLA, Prostate-UCLA dataset*.

In the WORC database, we obtained results ranging from weak discrimination for the Melanoma dataset (AUC = 0.56 [95% CI: 0.29–0.93], F1 = 0.48 [95% CI: 0.15–0.76]) to excellent discrimination for the Lipo dataset (AUC = 0.93 [95% CI: 0.77–1.0], F1 = 0.85 [95% CI: 0.67–1.0]) and the Desmoid dataset (AUC = 0.90 [95% CI: 0.79–0.98], F1 = 0.77 [95% CI: 0.57–0.92]).

For prostate datasets, results are reported separately for classification using features from either prostate or lesion masks. The discrimination was acceptable for both prostate masks (AUC = 0.73 [95% CI: 0.55–0.89], F1 = 0.73 [0.55–0.86]) as well as lesion masks (AUC = 0.72 [95% CI: 0.56–0.86], F1 = 0.68 [95% CI: 0.48–0.84]) in the PROSTATEx dataset, and moderate for prostate masks (AUC = 0.61 [95% CI: 0.51–0.71], F1 = 0.78 [95% CI: 0.72–0.74]) and lesion masks (AUC = 0.65 [95% CI: 0.55–0.74], 0.73 [95% CI: 0.67–0.80]) in the Prostate-UCLA dataset. Both prostate datasets were additionally validated using the other dataset, and their performance varied from AUC = 0.51 [95% CI: 0.41–0.61] for the PROSTATEx model using lesion masks evaluated in Prostate-UCLA to AUC = 0.77 [95% CI: 0.60–0.91] for the Prostate-UCLA using lesion masks evaluated in PROSTATEx.

The additional evaluation of the prostate MRI datasets for differentiation between clinically significant and clinically non-significant prostate cancer is presented in the Supplementary Table S2. For this challenging task, the results were worse than those for prostate cancer detection, with AUCs ranging from 0.40 [95% CI: 0.29–0.50] for the Prostate-UCLA dataset to AUC = 0.70 [95% CI: 0.33–0.97] for the PROSTATEx dataset, trained with prostate masks. The external validation results in this dataset showed AUCs in the range of 0.37 to 0.70 with high variability.

## Discussion

In this study, we introduced and validated a new open-source, interactive framework for reproducible radiomics research. The tool aids in selecting the optimal model for a given task, and the associated web application lowers the entry threshold for clinicians who want to contribute to the field of radiomics research and foster clinical translation.

We evaluated AutoRadiomics in six different classification tasks from the WORC database. It achieved consistently high AUCs in both cross-validation and the test set, in the direct comparison of our results vs. those reported in the original publication on the dataset (2): 0.93 vs. 0.83 for the Lipo dataset, 0.90 vs. 0.82 for the Desmoid dataset, 0.67 vs. 0.81 for the Liver dataset, 0.69 vs. 0.77 for the GIST dataset, 0.75 vs. 0.68 for the CRLM dataset, 0.56 vs. 0.51 for the Melanoma dataset. That means, our framework achieved comparable results, higher in 4/6 tasks, and lower in 2/6 tasks. We also evaluated our framework in two public prostate MRI datasets, achieving AUCs in the range of 0.61–0.73 for internal validation, and 0.51–0.77 for external validation. Those results prove that AutoRadiomics can be successfully applied off-the-shelf and achieve competitive results with its automatic configuration. We believe the differences between ours and previously reported results may be largely explained by the relatively small sample sizes, different data splitting, and differing choice of classifiers. It has to be noted, however, that, similarly to Starmans et al. (2), we achieved best results for the Lipo dataset, and worst for the Melanoma dataset, which suggests both approaches have converged to an optimal solution.

Quantitative evaluation of disease patterns in medical images which are invisible to the human eye has shown diagnostic potential in multiple retrospective studies, but large-scale clinical validation and adoption are still missing (22). We believe that an accessible toolkit for exploratory data analysis and a standardized workflow is a key component in developing the field toward clinical translation. With this in mind, we released AutoRadiomics as an intuitive open-source framework that structures the radiomics workflow and makes it more accessible and reproducible.

Recent advances in automated ML have the potential to empower healthcare professionals with limited data science expertise (23). Inspired by those breakthroughs, new platforms for ML applied to medical imaging have recently been introduced, such as WORC (2), which focuses on automatic construction and optimization of the radiomics workflow. While this platform also provides an automated solution and is very extensive in scope, AutoRadiomics sets itself apart with its interactive web interface, state-of-the-art tooling, and additional utilities (i.e., for visualization and segmentation).

With our web application, we hope to shift the focus from metrics to interpretability, which is achieved through comprehensive visualizations and radiomics maps. We would like to point out a few scenarios, where AutoRadiomics could be especially helpful: (1) for clinicians exploring their dataset using the embedded web application to gain quick insight into their data, (2) for researchers using Python for radiomic analyses, who want to complement their current workflow or add a benchmark or reference standard, (3) for an inter-institutional collaboration as means of facilitating results sharing and workflow reproducibility.

Currently, our framework can be used only for binary classification tasks, which limits its applicability. We are planning to extend it in the future to handle multiclass classification, regression tasks, and survival data. Furthermore, some processing steps such as automatic segmentation using deep learning require GPU capability, which is why it is not integrated into our framework and only the code for performing segmentation with a nnU-Net can be generated. AutoRadiomics does not require a powerful GPU and a modern personal computer is enough to run it. One should also keep in mind that the results of any optimized model have to be considered with caution and no abstraction layer (such as our web application) may replace true expert domain knowledge.

In conclusion, we herein presented AutoRadiomics, a framework for intuitive and reproducible radiomics research. We described its key features as well as the underlying architecture, and we discuss its most promising use cases. Finally, we validated it extensively in eight public datasets to show its consistently high performance in various and diverse classification tasks. We believe that AutoRadiomics may help to improve the quality and reproducibility of future radiomics studies, and, through its accessible interface, may bring those studies closer to clinical translation.

## Data Availability Statement

The original contributions presented in the study are included in the article/Supplementary Material, further inquiries can be directed to the corresponding author/s.

## Ethics Statement

Ethical review and approval was not required for this study in accordance with the local legislation and institutional requirements.

## Author Contributions

Study conception: BB. Radiomics analysis: PW. Statistical analysis: PW and FL. Draft writing: PW and BB. Manuscript edition: FL and TB. All authors contributed to the article and approved the submitted version.

## Funding

This work was supported by the Deutsche Forschungsgemeinschaft (DFG, German Research Foundation) (SPP 2177 to BB).

## Conflict of Interest

The authors declare that the research was conducted in the absence of any commercial or financial relationships that could be construed as a potential conflict of interest.

## Publisher's Note

All claims expressed in this article are solely those of the authors and do not necessarily represent those of their affiliated organizations, or those of the publisher, the editors and the reviewers. Any product that may be evaluated in this article, or claim that may be made by its manufacturer, is not guaranteed or endorsed by the publisher.
