# Supplementary material for: AutoRadiomics: A Framework for Reproducible Radiomics Research
Source: Front Radiol. 2022 Jul 7;2:919133. doi: 10.3389/fradi.2022.919133 (PMC10365084; doi:10.3389/fradi.2022.919133)
Supplement: Supplementary file 1 [file Data_Sheet_1.docx]

# Supplementary Material

**
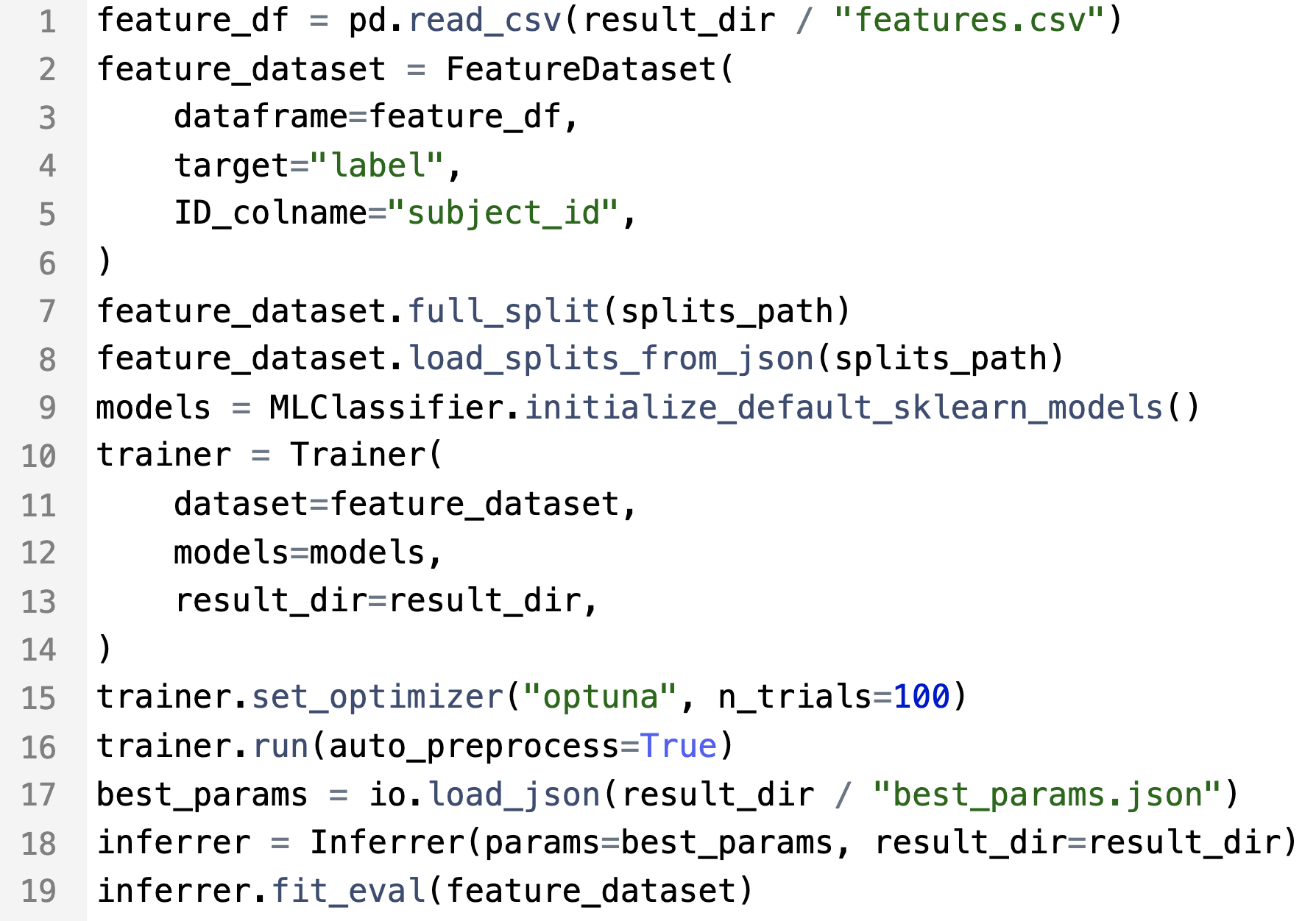
**

**Figure S1.** Example of minimal Python code executing the radiomics experiment.

**Table S2.** Additional evaluation for the prostate MRI datasets for differentiation between clinically significant and clinically non-significant prostate cancer.

| **Dataset** | **AUC** | | **F1** | **Sensitivity** | **Specificity** |
| --- | --- | --- | --- | --- | --- |
|  | 5-fold CV | test | test | test | test |
| Prostate masks: |  |  |  |  |  |
| PROSTATEx | 0.73 ± 0.08 | 0.70 [0.33-0.97] | 0.68 [0.47-0.84] | 0.65 [0.41-0.86] | 0.75 [0.54-0.94] |
| UCLA | 0.48 ± 0.07 | 0.40 [0.29-0.50] | 0.13 [0.05-0.23] | 0.07 [0.02-0.13] | 1.0 [1.0-1.0] |
| PROSTATEx→UCLA* |  | 0.70 [0.61-0.78 | 0.77 [0.70-0.83] | 0.68 [0.60-0.76] | 0.69 [0.55-0.84] |
| UCLA→PROSTATEx |  | 0.48 [0.14-0.81] | 0.66 [0.40-0.87] | 0.57 [0.31-0.83] | 0.66 [0.2-1.0] |
| Lesion masks: |  |  |  |  |  |
| PROSTATEx | 0.67 ± 0.09 | 0.51 [0.23-0.79] | 0.56 [0.27-0.78] | 0.43 [0.18-0.71] | 0.84 [0.5-1.0] |
| UCLA | 0.56 ± 0.05 | 0.58 [0.46-0.70] | 0.58 [0.46-0.70] | 0.51 [0.40-0.62] | 0.70 [0.55-0.86] |
| PROSTATEx→UCLA |  | 0.55 [0.44-0.66] | 0.78 [0.71-0.85] | 0.83 [0.75-0.91] | 0.33 [0.18-0.48] |
| UCLA→PROSTATEx |  | 0.37 [0.08-0.70] | 0.77 [0.58-0.92] | 0.86 [0.64-1.0] | 0.17 [0.0-0.56] |

*Arrow denotes external validation of the model trained on PROSTATEx dataset in the Prostate-UCLA dataset

**Appendix S3.** Details on final pipeline configuration for each task.

For each of the eight datasets evaluated, parameters including optional feature selection, ML classifier and its parameters, were tuned in 5-fold cross-validation. The final configurations are described below:

| **Dataset** | **Lipo** | **Desmoid** | **Liver** | **GIST** | **CRLM** |
| --- | --- | --- | --- | --- | --- |
| Feature selection | Boruta | Boruta | Boruta | RFE | RFE |
| ML classifier | Random Forest | LogReg | LogReg | LogReg | LogReg |
| ML classifier parameters | n_estimators: 882,  max_depth: 2,  max_features: sqrt,  min_samples_leaf: 6,  min_samples_spllits: 3,  bootstrap: False | penalty: L1,  C:9.44 | penalty: L2,  C: 0.027 | penalty: L1,  C: 6.97 | penalty: L2,  C: 0.007 |
| **Dataset** | **Melanoma** | **PROSTATEx (prostate masks)** | **UCLA (prostate masks)** | **PROSTATEx (lesion masks)** | **UCLA (lesion masks)** |
| Feature selection | RFE | RFE | RFE | RFE | RFE |
| ML classifier | SVM | XGBoost | SVM | LogReg | LogReg |
| ML classifier parameters | kernel: rbf,  C: 0.03,  gamma: 0.17,  degree: 1.0 | lambda: 3.8,  alpha: 1.4,  colsample_bytree: 0.2,  subsample: 0.95,  booster: gbtree,  max_depth: 5  min_child_weight: 8,  eta: 3.71,  gamma: 0.0002,  grow_policy: depthwise | kernel: linear,  C: 0.05,  gamma: 0.11,  degree: 1.0 | penalty: L2,  C: 0.79 | penalty: L2,  C: 0.02 |
|  |  |  |  |  |  |

*RFE – recursive feature elimination, LogReg – logistic regression, SVM – Support Vector Machine
